# Measuring Serum Level of Ionized Magnesium in Patients with Migraine

**Published:** 2015

**Authors:** Farhad ASSARZADEGAN, Mostafa ASADOLLAHI, Hojjat DERAKHSHANFAR, Azam KASHEFIZADEH, Omid ARYANI, Mona KHORSHIDI

**Affiliations:** 1 Department of Neurology, Imam Hossein Hospital, Shahid Beheshti University of Medical Sciences, Tehran, Iran; 2 Emergency Department, Imam Hossein Hospital, Shahid Beheshti University of Medical Sciences, Tehran, Iran; 3 Neurologist, Tehran, Iran; 4 Neurologist, Special Medical Center, Tehran, Iran; 5 Medical student, Azad University, Faculty of Medicine, Yazd, Iran

**Keywords:** Headache, Migraine, Serum ionized magnesium

## Abstract

**Objective:**

Migraine is known as one of the most disabling types of headache. Among the variety of theories to explain mechanism of migraine, role of serum magnesium is of great importance. Serum magnesium, as a pathogenesis factor, was considerably lower in patients with migraine. We established this study to see if serum ionized magnesium, not its total serum level, was different in migraineurs from normal individuals.

**Materials & Methods:**

In this case control study, all participants were recruited from Neurology Clinic of Imam Hossein Hospital, Tehran, Iran. Ninety-six people were entered in the study, 48 for each of case and control groups. The two groups were matched by age and sex. Migrainous patients were selected according to the criteria of International Headache Society. Various characteristics of headache were recorded based on patients’ report. Controls had no history of migraine or any significant chronic headaches. Serum ionized magnesium level was measured in both of the case and control groups and the results were compared to each other. P value of <0.05 was considered as significant.

**Results:**

Case group consisted of 13 males, 35 females, and control group included 14 males, as well as 34 females. Mean age was 33.47± 10.32 yr for case and 30.45 ±7.12 yr for control group. Twenty-eight patients described the intensity of their headaches as moderate; 15 patients had severe and the 5 remainders had only mild headaches. Mean serum level of ionized Mg was 1.16± 0.08 in case group and 1.13± 0.11 in control group of no significant difference (P >0.05).

**Conclusion:**

Serum ionized magnesium, which is the active form of this ion, was not significantly different in migraineurs and those without migraine. This may propose a revision regarding pathogenesis of migraine and question the role of magnesium in this type of headache.

## Introduction

According to WHO statistics, headache is one of the most frequent disabling conditions encountered in medicine. Migraine is the most common headache with vascular origin, which affects about 18% of women and 6.5% of men. More than 90% of the migraineurs state a relative inability to do their daily activity and about 53% require complete resting during an attack (1). Regarding the high incidence and prevalence of migraine and its disabling nature, it seems crucial to detect the exact mechanism. This can guide us through better plans for disease management. Significance of magnesium (Mg) in pathogenesis of migraine has been credited in some studies and refused in others. Magnesium deficiency is much more prevalent in migraine sufferers than in healthy controls (2). Besides, based on insufficient data, magnesium has been used for migraine prophylaxis and treatment of acute migraine in clinic (3). This study was set up to challenge the role of Mg in migraine by measuring serum ionized Mg level, which is the active form of this ion, in migraine patients referred to Imam Hossein Hospital, Tehran, Iran and to compare the values with normal population.

## Materials & Methods

In this case control study, considering the parameters of P=0.50, α=0.05, d=0.10, the size of sample groups was estimated at least 96 people totally, 48 people for each of the case and control groups. The case group included 48 migraine patients recruited from Neurology Clinic of Imam Hossein Hospital, Tehran, Iran. In addition, 48 individuals were randomly selected from patients visited in hospital neurology clinics and entered the study as control group. In past medical history and physical examination, none of the participants of control group had migraine or any major neurologic problems. The two groups were matched by age and sex. All aspects of the study were explained to the participants and they filled a consent form. The study was approved by the Ethics Committee of Imam Hossein Hospital. Inclusion criteria for the case group were as follows: definite migraine diagnosis on the basis of International Headache Society (IHS) criteria (4); at least two monthly episodes of headache; a history of headache during the last 3 months; no headaches at the time of blood sampling or within the previous two days; fasting for at least 10 hours before the blood sample was taken; no history of migraine prophylactic drugs or Mg supplements consumption; and no history of underlying systemic diseases. Those patients fulfilled the inclusion criteria, were examined carefully and essential lab data (including CBC and serum electrolytes) were requested. In addition to the topographic information, obtained from both groups, participants of the case group also filled a questionnaire containing most of the required parameters related to migraine. Patients filled the questionnaire two times; once at the first visit and next at the time the laboratory results were ready. Items of the questionnaire included: type of migraine (with or without aura), type of aura (visual, sensory, motor, language), location of the headache, presence or absence of orbital pain, accompanying symptoms (photophobia, phonophobia, nausea and vomiting), family history of migraine and severity of headaches. Severity of headache was recorded as a quantitative score using visual analog scale (VAS) which classifies headaches as mild (0-3), moderate (4-6), and severe (7-10). Serum ionized Mg level was measured in both case and control groups using Ion Selective Electrode (ISE) method which eliminates the effect of serum free calcium levels on measured serum ionized Mg (5). Because depletion of Mg from hemolyzed red blood cells could falsely increase serum Mg level and disturb the results, hemolysis of the samples was prevented. Data were analyzed using SPSS version 17 software (Chicago, IL, USA). X2 and t-test were applied to analyze and compare data. P value <0.05 was determined as significant.

## Results

Each of the case and control groups contained 48 people. Distribution of age and sex was matched in two groups (P value < 0.05 for both items). Mean age was 33.47± 10.32 yr for case and 30.45 ±7.12 yr for control group. Case group consisted of 13 males, 35 females, and control group included 14 males as well as 34 females. Most of the migraineurs were female (35 people) and 41 (85.5%) had migraine without aura. Only seven patients (14.5%) experienced auras of which 5 were purely visual and 2 were a combination of visual and sensory type. Thirty-three patients had bilateral and 15 had unilateral headaches. Majority of patients reported more than one region for their headaches. Frontal and temporal were dominant locations in most cases (83.3% and 77%).Headache in occipital and vertex regions were next in frequency. Orbital pain, which is a common presentation of migraine, was reported by 34 patients. Most common symptoms accompanying headaches were phonophobia (44 people; 91.6%) and photophobia (41 people; 85.4%). Frequency of migraine accompanying symptoms is shown in Table 1. Twenty-eight patients (58.3%) had a history of the disease in their first degree relatives while 20 patients (41.7%) had no family history of migraine. Using VAS, 28 patients described the intensity of their headaches as moderate, 15 patients had severe and the 5 remainders had mild headaches. Mean serum level of ionized Mg was 1.16± 0.08 mEq/L in case group and 1.13± 0.11 mEq/L in control group. There was no significant difference on serum ionized Mg level between the two groups (P value > 0.05).

**Table 1:** Frequency of Symptoms Accompanying Headache. Each patient May Have More Than One Accompanying Symptom

**Symptom**	**Number**	**Percentage**
Photophobia	41	85.4
Phonophobia	44	91.6
Osmophobia	6	12.5
Nausea	32	66.6
Vomiting	10	20.8

## Discussion

Migraine seems to have a complex pathogenesis. To explain the mechanism of migraine several theories have been suggested. A phosphorylation oxidative defect, malfunction of intraneuronal voltage gated calcium channels, intracellular magnesium (Mg) deficiency or a combination of these may make the cells susceptible to spontaneous depolarization (6). Serum Mg has an influence over the serotonin receptors, synthesis and release of nitrous oxide, N-Methyl-D-aspartate (NMDA) receptor and some other neurotransmitters (7). Serum Mg level is lower in migraineurs. Sun and colleagues evaluated the role of Mg as an important intracellular element. They showed that Mg interacted with serotonin receptors and reduced the severity of migraine headache. They also showed that Mg took part in diffuse cortical depression, altered the release of some neurotransmitters and enhanced platelet aggregation. They suggested that prophylactic use of oral Mg or therapeutic administration of intravenous Mg during an episode of headache was a useful approach and reduced migraine attacks (8). Altura et al. stressed that recalcitrant headaches might be relieved by intravenous Mg sulfate (9). In menstrual migraine, ionized Mg level is decreased and the ratio of Ca/Mg level is increased. Therefore, serum Mg level may have a role in pathogenesis of menstrual migraine (10). Mishima et al. and Aloisi et al. also reported a lower serum Mg in migrainous patients (11, 12). Trauninger and colleagues concluded that there was no significant difference between migraineurs and normal individuals in both serum and urine Mg level although the average serum Mg was lower in patients with migraine (13). According to results of our study, serum level of ionized Mg, which is the active form of magnesium, was not significantly different in migraineurs from those without migraine. This is in contrast with the results of the study by Mauskop et al. (9). However, most of the other studies mentioned above had emphasized the role of total serum Mg. In this study ionized Mg level was measured instead of its total amount. This may propose a revision regarding pathogenesis of migraine and question the role of this ion in migraine. In conclusion, it seems that measurement of total serum Mg level is not sufficient to explain migraine mechanism, because only the ionized form of Mg has biologic effectiveness. Further investigation may be required in this issue.
